# Dermatological Presentation of *Strongyloides stercoralis* Infection in Two Elderly Italian Inpatients

**DOI:** 10.3390/pathogens13080658

**Published:** 2024-08-06

**Authors:** Giulia Gardini, Guenter Froeschl, Petra Rosa Puzzi, Silvia Gambino, Elke Maria Erne

**Affiliations:** 1 Department of Infectious Diseases, Provincial Hospital of Bolzano (SABES-ASDAA), Lehrkrankenhaus der Paracelsus Medizinischen Privatuniversität, 39100 Bolzano-Bozen, Italyelkemaria.erne@sabes.it (E.M.E.); 2 Division of Infectious Diseases and Tropical Medicine, University Hospital (LMU), 80802 Munich, Germany

**Keywords:** *Strongyloides stercoralis*, strongyloidiasis, skin lesions, *larva currens*

## Abstract

Persistent infections caused by *Strongyloides stercoralis* are probably underestimated in the elderly Italian population. This nematode is unique among helminths: it can last asymptomatically in the host for decades and may present with a broad range of clinical pictures upon reactivation. Misdiagnosis often occurs even when the clinical picture is suggestive. If undetected, this parasitosis can lead to serious consequences when hyperinfection occurs. Herein, we report two peculiar clinical cases of complicated strongyloidiasis with multiple skin lesions. The aim of our report is to lead clinicians to familiarize themselves with skin patterns and clinical features that can suggest a possible underlying strongyloidiasis.

## 1. Introduction

*S. stercoralis* infection is neglected both in low- and high-resource countries. In 2020, the World Health Organization (WHO) declared that *S. stercoralis* was not previously considered in STH (soil-transmitted helminthiases) prevention and control programs [1]. Ivermectin used in mass drug administration campaigns was directed against lymphatic filariasis and onchocerciasis. The 2030 targets for STH control programs by the WHO contain one part dedicated to the control of strongyloidiasis in children at risk living in high endemicity countries [1].

In 2017, the estimated prevalence of strongyloidiasis in Italy at the time was 0.03% (CI 0.01–0.04%) [2]. This overall low percentage might lead clinicians to underestimate the clinical relevance of *S. stercoralis*. However, when approaching migrants from endemic countries, international travellers, and elderly Italians, we should be aware that the prevalence of *S. stercoralis* infection may be much higher in these subgroups. When focusing on elderly Italians, cross-sectional studies have reported prevalences of 8% in outpatients with eosinophil count ≥ 500 cells/µL and 1% in outpatients with eosinophil count < 500 cells/µL [3], 28% in outpatients with eosinophil count ≥ 500 cells/µL [4], 14% in inpatients (half with eosinophilia and half without, with a higher risk for those with eosinophilia) [5], and 20% in inpatients with invasive bacterial infection of enteric origin [6]. These epidemiological findings suggest that Italy was probably endemic for strongyloidiasis before the 1950s, i.e., before sanitary domestic conditions improved for the general population and massive rural–urban migration commenced after World War II. The peculiarity of the parasite is that when the infection is acquired, it can last for decades without treatment. Therefore, elderly Italians even without a relevant travel history are at a higher risk of having this parasitosis.

The clinical patterns of the infection are heterogeneous. The skin, gastrointestinal tract, and respiratory apparatus are mainly involved. The most severe clinical manifestations appear when an immunosuppressive factor (e.g., administration of systemic corticosteroids even for a short period of time) [7] or condition (e.g., diabetes, advanced age, alcoholism, or solid organ or hematopoietic stem cell transplanted patients) [8,9,10] occur. In these cases, the larval production of the adult worm localized in the small intestine of the host has the opportunity to increase due to a deficient immunological control by the host. A variable extended number of larvae pass the intestinal barrier of the host and invades the sites that typically host the biological cycle of the parasite (the skin, gastrointestinal tract, and respiratory apparatus) or other organs when the number of passing larvae is high (e.g., the central nervous system) [11]. Sometimes, clinical status is worsened by concomitant bacterial gut translocation and subsequent invasive bacterial infection (e.g., meningitis) [12].

Cutaneous manifestations can be different:

The acute phase of the infection may involve:

- Transient localized edema or urticaria where the filariform larva (L3, up to 600 µm long) enters the intact skin of the host

The chronic phase of the infection may involve:

- *Larva currens* as a pathognomonic serpiginous urticarial running lesion (at least 1 cm/h) that appears mainly on the skin of the inferior abdomen, buttocks, or perineal area, caused by L3 larval passage through the dermal layer of the host [13];
- Petechiae and purpura on the abdomen [14,15,16];
- Other unspecific lesions with variable extension [16,17,18,19];
- Pruritus can accompany the above lesions or present alone [18,20].

The extent of the skin pattern relates to the intensity of larval proliferation (e.g., multiple diffuse lesions vs. single *larva currens*) and the immunological reaction to migrating larvae. Some skin patterns are pathognomonic for strongyloidiasis (*larva currens*), some are related to *S. stercoralis* dissemination (periumbilical purpura), and some are unspecific.

Herein, we present the case of two elderly Italian inpatients where the appearance of skin lesions contributed to the presumptive diagnosis of strongyloidiasis and to prompt initiation of anti-parasitic treatment, even before laboratory confirmation was available, possibly avoiding a potentially rapidly evolving and life-threatening hyperproliferative condition.

## 2. Case Report 1

In March 2022, we admitted an 82-year-old Italian patient for chronic obstructive pulmonary disease (COPD) exacerbation during paucisymptomatic SARS-CoV-2 infection. He was born in southern Italy, and during his working life, he worked as a railroad and quarry worker in Italy and northern Africa.

**Figure 1:**
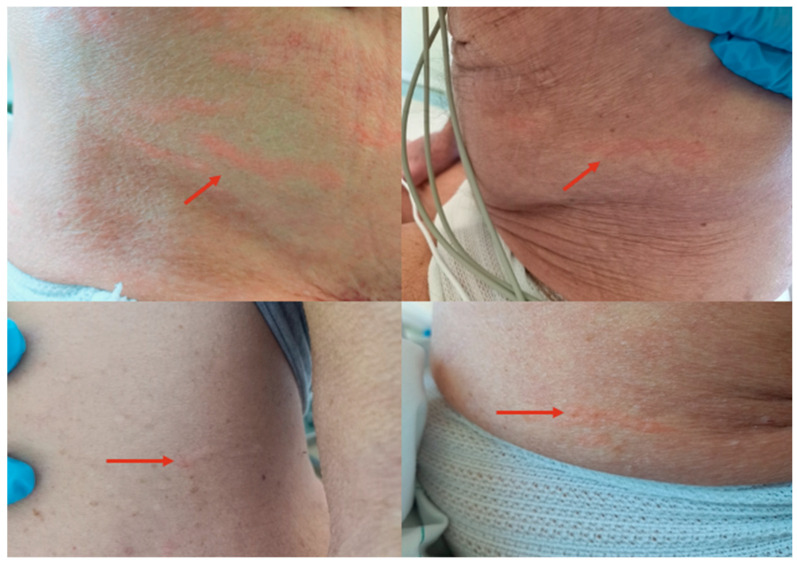
Disseminated *larva currens* on the inferior abdomen.

He was treated daily with 40 mg intravenous methylprednisolone. On the second day of therapy, he developed a persistent dry cough, increased necessity of oxygen support, and multiple mildly itchy cutaneous lesions on his abdomen (Figure 1). We recognized the skin lesions as disseminated *larva currens* and therefore pathognomonic for *S. stercoralis* hyperinfection even in the absence of eosinophilia.

We promptly treated him with oral ivermectin (200 µg/kg/day for two consecutive days with repeated treatment for two consecutive days after 2 weeks), and we obtained a rapid improvement in respiratory and skin manifestations shortly after the first ivermectin administration.

We decided to treat him immediately without microbiological confirmation for two reasons: *larva currens* is pathognomonic for *S. stercoralis* infection and the clinical picture suggested a possible life-threatening parasitic hyperinfection.

As part of our diagnostic method, we collected three stool samples on three consecutive days (only one was collected before ivermectin administration) for direct microscopy examination (performed in our laboratory of microbiology) and one blood sample for highly sensitive (93.9%) and specific (92.2%) in-house IFAT serology (performed in the Laboratory of Microbiology, Don Calabria Hospital, Negrar, Verona, Italy) [21]. Direct microscopy examination of the stools did not detect *S. stercoralis* larvae or other parasitic elements, but the serology was positive with a titer of 1:80 (reference < 1:20).

## 3. Case Report 2

At the end of February 2023, we admitted a 90-year-old woman with clinical suspicion of bacterial meningitis. She was born in southern Italy, later as an adult she moved to Bolzano (South Tyrol Region, Italy), and during her life, she worked as a housewife. In the last few years, she had developed Alzheimer’s dementia, and for this reason, she stayed in a long-term healthcare facility in Bolzano.

Lumbar puncture showed cloudy cerebrospinal fluid (CSF) and a chemical–physical pattern compatible with the clinical suspicion. She was started on 12 g of ampicillin daily with intravenous continuous infusion and 2 g of intravenous ceftriaxone bid. The cultures of CSF were negative, but multi-sensitive *Klebsiella pneumoniae* was isolated in blood cultures. We stopped ampicillin but continued 2 g of intravenous ceftriaxone bid.

**Figure 2:**
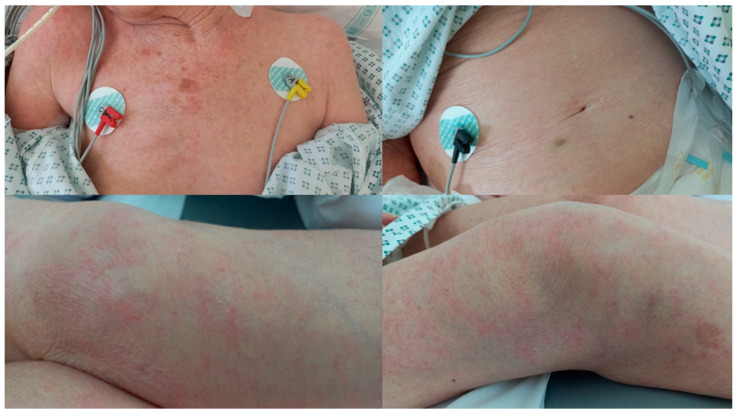
Diffuse exanthema in *S. stercoralis* infection.

On the 10th day of antibiotic treatment, she developed diffuse exanthema (Figure 2) and mild eosinophilia (550 cells/µL). The pre-existing advanced dementia did not permit us to understand if the skin lesions were itchy. She had no history of allergies or hematologic diseases. The co-existence of an invasive bacterial infection of enteric origin, eosinophilia, and exanthema led us to suspect underlying strongyloidiasis. We asked for a serology test to be performed (in-house IFAT serology performed in the Laboratory of Microbiology, Don Calabria Hospital, Negrar, Verona, Italy) as well as stool microscopy.

After three days, eosinophilia worsened, increasing by over 3000 cells/µL, and we decided to empirically administer 200 µg/kg/day of ivermectin as a single dose through a nasogastric tube while still waiting for serological results from Verona. Unfortunately, at that time, we were unable to collect a stool sample due to the patient having constipation.

One week later, she completed the 3-weeks intravenous antibiotic therapy for meningitis. Her neurological status still appeared to be intensively altered, while her vital signs were normal and inflammation parameters almost negative (procalcitonin 0.09 ng/mL; C reactive protein 3.5 mg/dL). At this point, she presented a white blood cell count of 49,870 cells/µL with 72.3% eosinophils and we suspected an underlying hematologic disease. The patient appeared to be suffering and showed non-compliant behavior. Food and fluids were given through the nasogastric tube. In agreement with the relatives, we finally decided not to go further with diagnostic research; only symptomatic therapy was maintained and she was re-transferred to the long-term healthcare facility.

Ten days later, we received a positive result of *S. stercoralis* serology with a titer of 1:640 (reference < 1:20).

We contacted the staff of the long-term healthcare facility who reported a slow but progressive improvement in the general status of the patient. She had begun to eat spontaneously and could stand with support. To ascertain the adequacy of single-dose ivermectin treatment, we searched for the helminth in the CSF (1 mL of CSF at −20 °C collected upon admission was still available) and we asked to collect stool samples. Moreover, the blood count was repeated.

Direct microscopy and molecular detection in CSF gave negative results. Stool samples were not available. Three months after ivermectin administration, the patient appeared to have clinically recovered from the acute event and her eosinophil count decreased to 990 cells/µL (16.9%). We decided not to administer further doses of ivermectin but to maintain the patient in follow-up to ascertain the normalization of eosinophils and the decrease in serology titer in 6–12 months. The patient’s relatives preferred not to go further with our follow-up considering age, comorbidities, and her improved clinical condition.

## 4. Discussion

The first case reported is peculiar in some aspects. Good-quality images of *larva currens* are infrequent in the literature because these lesions move and disappear in a short period of time (*larva currens* means larva that runs). The patient developed an *S. stercoralis* hyperinfection after a single dose of 40 mg of intravenous methylprednisolone. The absence of larval detection in the stool sample confirms the intermittent and fluctuant emission of larvae during chronic strongyloidiasis, even during hyperinfection when a great number of larvae in stage L3 pass through the intestinal barrier [22,23,24]. Empirical treatment should be considered in highly suggestive cases when available diagnostic methods have low sensitivity (direct methods applied to stool samples) or the results take time (in our case, serology performed in another hospital).

The second case may represent an example of a form of strongyloidiasis with accompanying bacterial transmigration through the intestinal wall, as has been newly hypothesized and described in a recent article [6]. Invasive bacterial infection of enteric origin represents the first “alarm signal” to consider an underlying strongyloidiasis. Multiple skin lesions and peripheral eosinophilia increase the positive predictive value of this diagnosis. We hypothesize that chronic strongyloidiasis may facilitate invasive bacterial infection of enteric origin even in cases of low-intensity larval migration outside the intestinal lumen and through immunomodulation [6], which means that phenomena of bacterial transmigration have to be expected in cases without signs of larval hyperinfection or dissemination as well. A standard regimen of treatment for this form of low larval yet transmigrating infestation has not been established, and the prognosis if left untreated is not known, not the least as we have to assume a high rate of unrecognized cases. In this case, the suspicion was based on three factors: bacterial enteric sepsis, skin lesions, and peripheral eosinophilia. Our patient responded to a single dose of 200 µg/kg ivermectin, as currently recommended for immunocompetent patients without hyperinfection [25]. There were no signs or reasons to suspect immunosuppression in the patient, except for supposed immunocompromised status due to older age. We did not have the opportunity to ascertain the eradication of the parasite through the monthly control of eosinophil count and the repetition of serology in 6–12 months after treatment.

## 5. Conclusions

Clinicians should be aware of the heterogenous cutaneous manifestations of chronic strongyloidiasis when approaching a patient with epidemiological risk factors for this parasitosis (e.g., elderly Italians, migrants, and international travelers). Early clinical suspicion of complicated forms and subsequent prompt empirical treatment is fundamental in settings where highly sensitive diagnostic tools are not available or have a long reporting time.

## Data Availability

The original contributions presented in the study are included in the article. Further inquiries can be directed to the corresponding author.
